# Impact of Purge Regime and Reactor Volume on ALD ZnO and ZrO_2_ Growth: From Structural Properties to Applications

**DOI:** 10.3390/ma19081556

**Published:** 2026-04-13

**Authors:** Lukasz Wachnicki, Sylwia Gieraltowska

**Affiliations:** Institute of Physics of the Polish Academy of Sciences, Aleja Lotnikow 32/46, 02-668 Warsaw, Poland; lwachn@ifpan.edu.pl

**Keywords:** ALD, ZrO_2_, ZnO, purge mode, dynamic vacuum, static vacuum, XRD, AFM

## Abstract

ALD is a precise thin-film deposition technique based on self-limiting surface reactions. A crucial stage in each ALD cycle is the purge step, which removes excess precursor molecules and reaction by-products from the reactor chamber, preventing uncontrolled gas-phase reactions that could degrade film quality. Despite its fundamental importance, the impact of purge dynamics on film growth and structure remains insufficiently explored. ZnO and ZrO_2_ films were deposited in reactors with different effective chamber volumes (47 and 470 cm^3^), enabling a systematic study of gas residence time effects. Our results demonstrate that the purge mode—dynamic versus static vacuum—strongly affects the growth behavior, crystallinity, and surface morphology of ALD oxides. Dynamic purging leads to smoother, more uniform, and better-crystallized films, whereas static exposure results in lower structural and morphological quality, particularly for ZrO_2_. Importantly, these results demonstrate that purge-mode engineering provides a powerful and cost-effective route for tailoring oxide film structure without altering the precursor chemistry or deposition temperature. To validate the practical integration of these optimized films, functional phosphor and LED structures were fabricated, confirming that the controlled microstructure is well-suited for optoelectronic applications. This approach also offers new possibilities for controlling film properties in sensors and catalysts.

## 1. Introduction

Atomic Layer Deposition (ALD) is a widely used thin-film deposition technique based on self-limiting surface reactions, offering excellent thickness control, high uniformity, and outstanding conformality [1,2,3]. These features make ALD particularly attractive for applications in microelectronics, optoelectronics, photonics, and advanced functional coatings. However, achieving high film quality in ALD depends not only on the choice of precursors and deposition temperature, but also critically on the dynamics of gas transport and the removal of reaction by-products during the process [1,4]. A key step in every ALD cycle is the purge step, whose role is to remove excess precursor molecules and reaction products from the reactor chamber and to prevent parasitic gas-phase reactions. Inadequate purging leads to precursor overlap and extended residence times, which may deteriorate film uniformity, surface morphology, and structural quality [3,5]. Despite its fundamental importance, the influence of purge dynamics on growth kinetics, microstructure, and crystallinity of ALD-grown films has received relatively limited attention, especially in relation to reactor volume and gas residence effects.

In typical ALD reactors, different purge strategies can be employed. In the static purge vacuum mode (SPVM), the pumping system is closed during precursor exposure, allowing the precursor to remain in the reactor volume for a longer time and increasing its partial pressure near the substrate surface. In contrast, in the dynamic purge vacuum mode (DPVM), continuous pumping and carrier gas flow are applied, ensuring faster and more efficient removal of residual species and better separation of successive ALD half-reactions. These two approaches are expected to result in different growth regimes, ranging from nearly ideal, self-limited ALD to conditions with an increased contribution of non-ideal growth that may resemble chemical vapor deposition (CVD)-like processes [1,3], as the precursor may not fully escape the features it has diffused into. However, a direct comparison of their impact on film growth and structure is still lacking. Oxide materials such as ZnO and ZrO_2_ were deliberately selected as model systems for this study for several complementary reasons. Both materials are technologically important and widely used in ALD-based applications: ZnO is a well-known wide-bandgap semiconductor employed in optoelectronics, transparent electronics, and sensors [6,7,8], whereas ZrO_2_ is a key high-k dielectric and a functional oxide used in photonic and phosphor-related structures [4,9,10]. Therefore, understanding how process parameters affect their growth is directly relevant for practical applications. At the same time, ZnO and ZrO_2_ differ significantly in their surface chemistry, precursor reactivity, and growth mechanisms in ALD. ZnO typically exhibits relatively robust and reproducible growth behavior [6,7,11], while ZrO_2_ is known to be more sensitive to process conditions, such as temperature and precursor residence time, showing a transition from amorphous to crystalline phases with increasing temperature [4,9,10]. This contrast makes them ideal complementary test systems for evaluating how purge dynamics influence growth per cycle, surface morphology, and crystallinity. Furthermore, both materials can be deposited over a wide temperature range, from low-temperature regimes suitable for temperature-sensitive substrates to higher temperatures that promote crystallization and improved structural quality [4,6,9]. This allows a direct comparison between low- and high-temperature growth regimes and enables assessment of how purge-mode engineering and temperature optimization jointly control film structure and conformality. As demonstrated in our previous reports [12,13], these materials have been successfully integrated into various functional applications, in which their structural integrity is a critical factor influencing device performance. Importantly, in most previous studies the film structure in ALD has been primarily tuned by changing the precursor chemistry or the deposition temperature [1,4,6,9]. In contrast, we present that purge-mode engineering—independent of precursor chemistry or deposition temperature—is a cost-effective strategy for tailoring the microstructure, morphology, and crystallinity of oxide thin films. This approach treats the purge step not only as a technical requirement, but also as an active process parameter for deliberate microstructure control.

This work presents a systematic study of the impact of purge mode on the growth, structure, and morphology of ZnO and ZrO_2_ thin films deposited by ALD. Two purge regimes—DPVM and SPVM—were compared in reactors with different effective chamber volumes (47 and 470 cm^3^), enabling an assessment of gas residence and scaling effects. The oxide films were characterized by X-ray diffraction (XRD) and atomic force microscopy (AFM) to correlate growth conditions with crystallinity and surface morphology, while growth per cycle (GPC) is used to analyze the growth kinetics. By linking process parameters with microstructure and film quality, this study showed that purge-mode engineering provides a simple and cost-effective route to tailor highly conformal oxide thin films without changing precursor chemistry [1,4,5]. These findings are crucial for the development of functional applications, enabling the successful integration of optimized ALD oxides into phosphor and light-emitting diode (LED) structures.

## 2. Materials and Methods

### 2.1. Thin Film Deposition

The ZnO and ZrO_2_ thin films were deposited using the atomic layer deposition system in a reactor operated under two purge regimes: dynamic purge vacuum mode and static purge vacuum mode. In the DPVM approach the pumping system remains active and the purge step is performed under continuous carrier gas flow combined with pumping. This dynamic purge ensures more efficient and faster removal of residual precursor molecules and reaction products from the reactor. In contrast, in the SPVM approach, during the precursor exposure step the pumping system is closed, which allows the precursor to remain in the reactor volume for an extended time and increases its partial pressure near the substrate surface. After the exposure step, the pump is opened and the excess precursor molecules and reaction by-products are evacuated from the reactor. Oxide layers were grown on (100)-oriented n-Si substrates using a Savannah-100 reactor from Ultratech/CNT (Veeco, San Jose, CA, USA). The ALD processes were performed at low temperatures of 80 °C for ZnO and 85 °C for ZrO_2_, yielding films approximately 150 nm thick, and at relatively high temperatures of 300 °C for ZnO and 240 °C for ZrO_2_, yielding films approximately 200 nm thick. Deposition temperatures below 100 °C were specifically optimized to ensure high packing density and the formation of homogeneous, relatively flat layers for each compound, as detailed in our previous works [10,11]. These films were suitable for comparing the structural and morphological properties of layers deposited in static versus dynamic vacuum purge modes during the ALD process. In contrast, elevated temperatures above 200 °C promoted enhanced crystallinity of the films, which translated into improved structural quality and functional properties, making them more suitable for applications in phosphor structures and n-i-p heterostructure diodes, as previously reported [12,13].

Furthermore, the film thicknesses (far above 100 nm) were deliberately selected to ensure a sufficient signal-to-noise ratio in X-ray diffraction measurements and surface with well-defined features in atomic force microscope analysis, enabling reliable phase identification, assessment of the degree of crystallinity, and analysis of structural and morphological parameters. Depositions were carried out in reactors with effective chamber volumes of 47 and 470 cm^3^. Each ALD cycle consisted of precursor pulsing, purge or exposure, oxidant pulsing, and a final purge. In SPVM, exposure steps followed precursor pulses, whereas in DPVM all purges were performed under continuous vacuum pumping. Nitrogen gas with a purity of 99.9999% was used as the carrier and purge gas. The growth parameters, including precursor pulse times, purging times after precursor pulses and deposition temperatures, were kept identical for both purge modes for each selected oxide. The films were grown using sequential pulsing of the metal precursor and the oxygen precursor in both reactor volumes, with purging periods of 10 s and 20 s, respectively. To ensure self-saturated and homogeneous deposition throughout the entire chamber volume, the optimum precursor pulse times were kept in the millisecond range. However, scaling up to the larger 470 cm^3^ chamber required a two-fold increase in pulse durations compared to the 47 cm^3^ reactor to maintain saturation. To implement the SPVM, an additional ‘wait time’ (also referred to as exposure time) of 1 s was introduced immediately after the precursor pulse. During this interval, the stop valve remains closed, allowing the precursor to diffuse thoroughly across the substrate surface and into the sample structure. The duration of a complete ALD cycle for oxide deposition was approximately 30 s. Precise thickness control was achieved because a constant amount of film material was deposited in each cycle, and the film thickness scaled with the number of ALD cycles. The oxide film thickness was performed by spectroscopic reflectometry using a NanoCalc 2000-UV-VIS system (Mikropack GmbH, Ostfildern, Germany). ZnO films were grown using zinc and oxygen precursors as diethylzinc (DEZ, ≥52 wt. % Zn basis, Zn(C_2_H_5_)_2_, Sigma-Aldrich, Merck, Darmstadt, Germany) and deionized water, while ZrO_2_ layers were deposited from zirconium and oxygen precursors as tetrakis(dimethylamido)zirconium(IV) (TDMAZ, ≥99.99%, trace metals analysis, Zr[(CH_3_)_2_N]_4_, Sigma-Aldrich, Merck, Darmstadt, Germany) and deionized water. The oxides were synthesized via double-exchange chemical reactions:

Zn(C_2_H_5_)_2_ + H_2_O → ZnO + 2C_2_H_6_

Zr[(CH_3_)_2_N]_4_ + 2H_2_O → ZrO_2_ + 4HN(CH_3_)_2_

**Figure 1:**
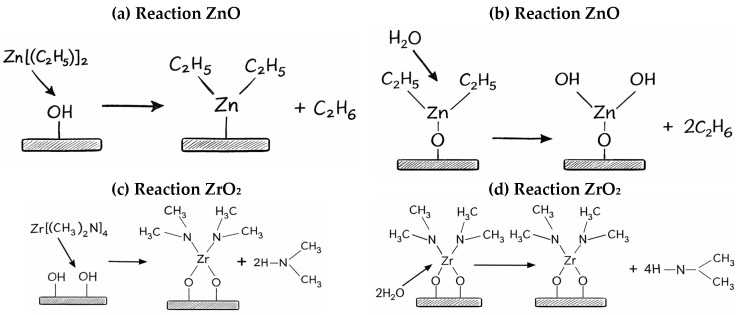
Schematic reactions of the ALD cycle: (**a**,**c**) metallic precursor pulse and inert gas purging; (**b**,**d**) oxygen precursor pulse and final inert gas purge in the dynamic purge vacuum mode and the static purge vacuum mode for ZnO (**a**,**b**) and ZrO_2_ (**c**,**d**) layers on substrates.

These reaction schemes occurring on the substrate during the ALD process are illustrated in Figure 1, taking into account both dynamic and static vacuum purge modes.

### 2.2. Structural and Morphological Characterization

The structural properties and crystallographic orientation of the layers deposited on silicon substrates were investigated by XRD. The measurements were carried out using an X’Pert Materials Research Diffractometer (Malvern Panalytical Ltd., Malvern, UK) equipped with an X-ray mirror and a two-bounce monochromator in the incident beam path. The diffracted signal was recorded using a two-dimensional solid-state X-ray detector (PIXcel). Phase identification was performed using the ICDD Powder Diffraction File database, with reference patterns for ZnO (PDF Card No. 00-005-0664) and ZrO_2_ (PDF Cards No. 00-021-1498 and 00-050-1089). The surface morphology of the oxide layers was characterized by AFM using a Bruker Dimension Icon system (Billerica, MA, USA) operated in PeakForce Tapping mode. Topographical data were acquired using high-resolution silicon nitride probes with a nominal tip radius of 2 nm. This imaging mode was selected to ensure precise force control and to minimize lateral forces during scanning [14]. The surface morphology of the ZnO and ZrO_2_ layers was characterized by evaluating their roughness, particle (grain) size, and particle density. Specifically, the root-mean-square (RMS) roughness, characteristic particle dimensions (height and width), and maximum height difference (R_max_) were extracted. Furthermore, the grain density was calculated based on a specific threshold height analysis performed on representative scan areas [15,16]. The topographical parameters were derived from AFM height measurements acquired over scan areas of 10 µm × 10 µm for the ZnO layers, and both 2 µm × 2 µm and 10 µm × 10 µm for the ZrO_2_ samples. The RMS roughness and R_max_ values for ZrO_2_ were determined from the 10 µm × 10 µm scans, whereas the remaining morphological parameters were extracted from the smaller 2 µm × 2 µm areas, which provided a more detailed representation of the surface topography. The RMS roughness of the Si substrate was measured at approximately 1 nm.

**Figure 2:**
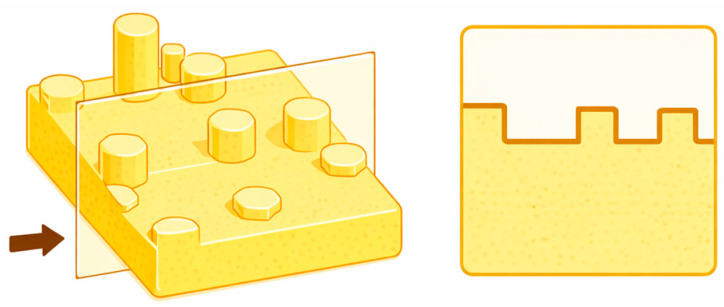
Schematic cross-sectional profile (**right**) extracted from the AFM topography data (**left**, location marked with an arrow), illustrating the methodology for determining the characteristic height and width of the surface particles (grains) on the deposited layers.

The characteristic grain size was defined by the typical height and width of the surface features, as determined from the cross-sectional profiles illustrated in Figure 2. The cross-sectional profiles reported in this study were taken along the central axes (5 µm and 1 µm) of the 10 µm × 10 µm and 2 µm × 2 µm AFM scans, respectively. The parameter R_max_ was defined as the maximum vertical distance between the highest and lowest data points within the analyzed image. The RMS roughness was defined as the root mean square average of height deviations from the mean image data plane, expressed as:

$$\mathrm{RMS}=\sqrt{\frac{1}{N}\sum_{i=1}^{N}z_{i}^{2}}$$

where *z_i_* is the height value of the *i*-th pixel and *N* is the total number of data points in the analyzed AFM image [14].

**Figure 3:**
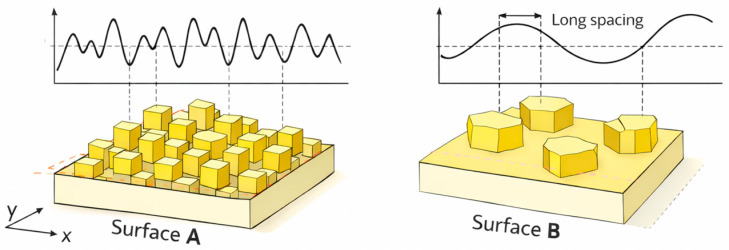
Schematic comparison of two surfaces with different spatial distributions of surface features. Surface A exhibits densely packed, high-frequency roughness, while Surface B shows sparsely distributed, low-frequency features with long lateral spacing. Although the height profiles differ, both surfaces may exhibit similar RMS roughness.

It should be noted that two surfaces may exhibit the same RMS roughness value despite having significantly different spatial distributions of surface features. For example, a surface characterized by high-frequency height variations and another surface with lower-frequency but similar amplitude variations may yield identical RMS values (Figure 3). In terms of RMS roughness, both surfaces are therefore equally rough, and to distinguish between such surfaces, additional parameters, such as particle analysis, must be considered. This analysis was performed using a threshold height, which is a critical parameter for identifying individual surface features. The threshold height was determined from the particle size (depth) histogram, which was fitted using a Gaussian distribution, allowing the determination of a characteristic average grain height. Based on this analysis, the average grain density was calculated as the total number of identified particles per unit area (µm^2^) for the selected threshold height. This method is particularly suitable for enabling more accurate and reproducible identification of particles.

## 3. Results and Discussion

The influence of the purge strategy on the growth behavior is directly reflected in the growth per cycle values summarized in Table 1. For both ZnO and ZrO_2_ films, and for investigated reactor volumes, the GPC obtained in SPVM is systematically higher than that measured in DPVM. This behavior can be explained by the fundamentally different gas transport conditions in both modes. In SPVM, closing the pumping system during exposure extends gas residence time, which may promote re-adsorption and secondary reactions. This can trigger higher GPC values. Conversely, DPVM utilizes continuous pumping and carrier gas flow to effectively remove precursors and by-products.

Although this leads to a slightly lower GPC, it provides growth control and eliminates non-specific gas-phase reactions [3,17]. Importantly, the comparison between the two reactor chamber volumes shows that, within the experimental uncertainty, the GPC values are essentially identical for a given material and purge mode. On the contrary, the use of the smaller chamber offers clear practical advantages, such as lower precursor consumption and a higher confidence in achieving uniform deposition throughout the entire chamber volume. From a technological point of view, this makes the small-volume reactor a more efficient and economically attractive solution without compromising film quality.

The higher GPC of ZrO_2_ compared to ZnO originates from differences in precursor chemistry and surface reaction mechanisms (Figure 1). In ALD, the growth per cycle is determined not only by the surface coverage, but also by the amount of material deposited per reactive site. The zirconium precursor TDMAZ is a multifunctional molecule containing four -N(CH_3_)_2_ ligands, which enables efficient reaction with surface -OH groups [1]. Although the larger molecular size of TDMAZ may introduce steric hindrance effects that limit the maximum surface coverage, each chemisorption event results in the incorporation of a relatively large amount of material due to the high atomic mass of zirconium and the formation of a ZrO_2_ unit. As a result, the mass deposited per cycle can be higher for ZrO_2_ than for ZnO, even if the surface site density or steric accessibility differs. The observed higher GPC of ZrO_2_ is therefore attributed to the combined effect of precursor chemistry and the higher mass contribution per adsorption event, rather than to film density alone. This interpretation is consistent with the known differences in ALD growth mechanisms between metal oxides based on different metal centers [18,19].

**Figure 4:**
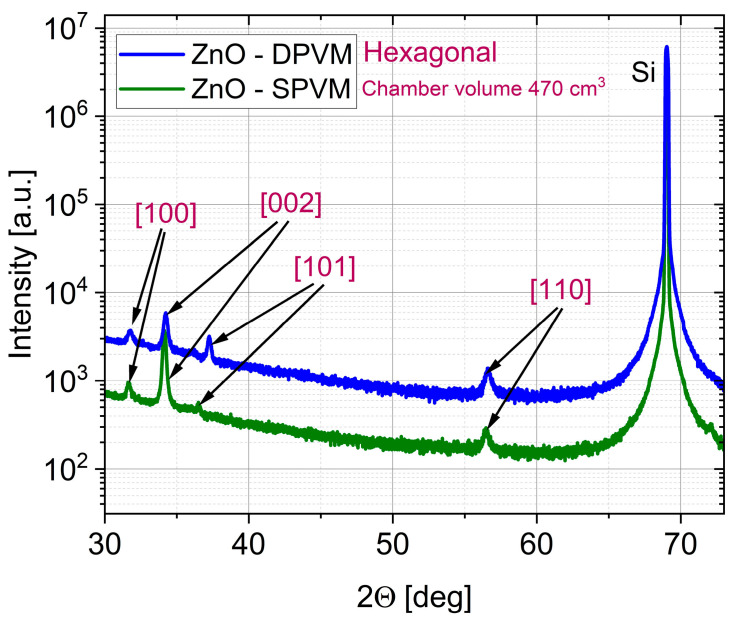
XRD patterns of ZnO thin films deposited at temperature lower than 100 °C by ALD in DPVM and SPVM in a reactor with a chamber volume of 470 cm^3^.

**Figure 5:**
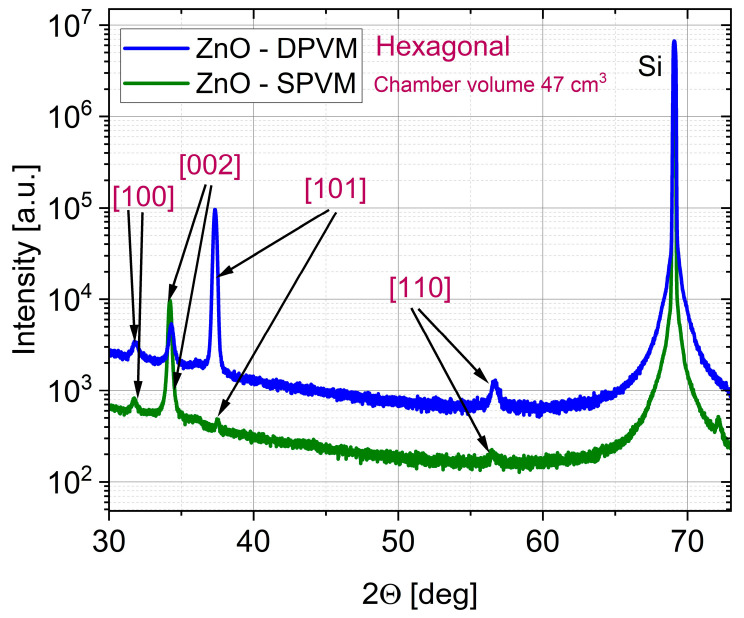
XRD patterns of ZnO thin films deposited by ALD at temperature lower than 100 °C in DPVM and SPVM in a reactor with a chamber volume of 47 cm^3^.

**Figure 6:**
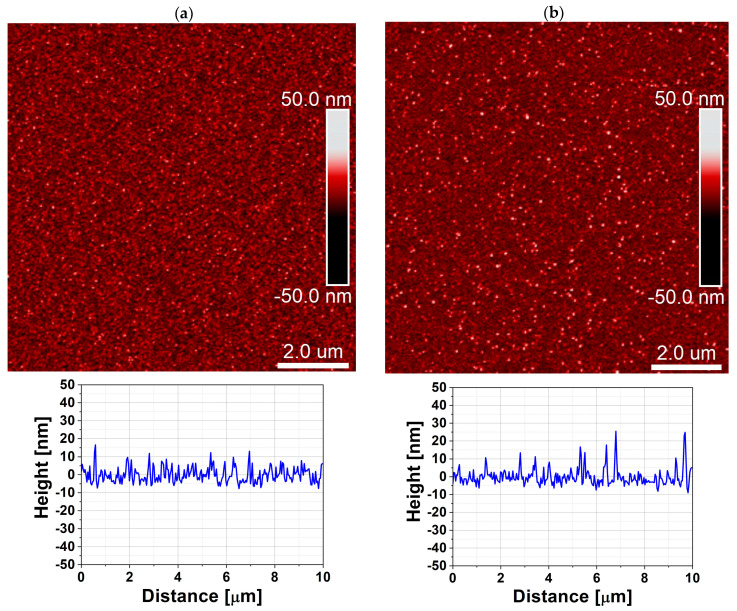
AFM surface topography with cross-sectional profile of ZnO thin films deposited at temperature lower than 100 °C by ALD in a reactor with a chamber volume of 470 cm^3^ using (**a**) dynamic purge vacuum mode (DPVM, RMS = 4 nm) and (**b**) static purge vacuum mode (SPVM, RMS = 4 nm). The scan area is 10 µm × 10 µm.

**Figure 7:**
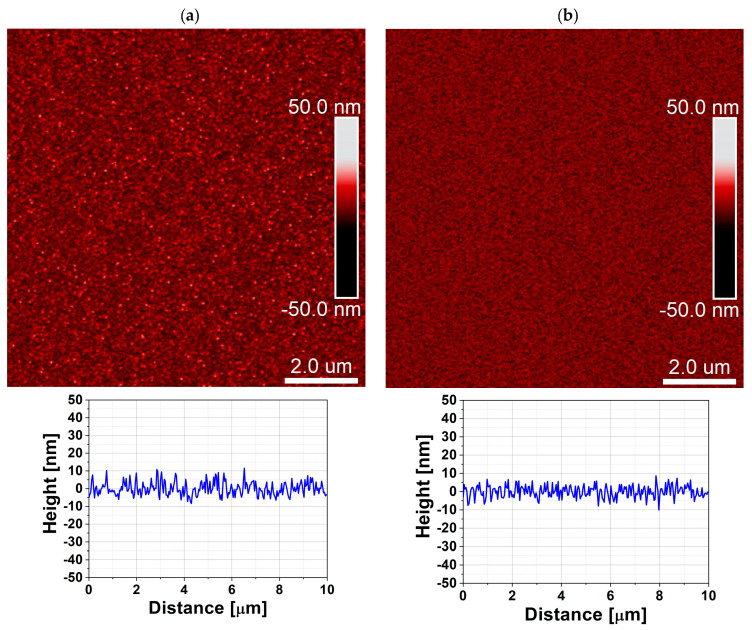
AFM surface topography with cross-sectional profile of ZnO thin films deposited at temperature lower than 100 °C by ALD in a reactor with a chamber volume of 47 cm^3^ using (**a**) dynamic purge vacuum mode (DPVM, RMS = 4 nm) and (**b**) static purge vacuum mode (SPVM, RMS = 3 nm). The scan area is 10 µm × 10 µm.

Figure 4 and Figure 5 present the XRD patterns of ZnO thin films deposited in DPVM and SPVM for both reactor volumes (470 and 47 cm^3^, respectively). In all cases, the diffraction peaks, including the (10.0), (00.2), (10.1), and (11.0) planes, correspond to the hexagonal wurtzite structure of ZnO. However, clear differences in structural ordering are observed between the two regimes. Films grown in DPVM show higher peak intensities and a better signal-to-noise ratio, indicating improved crystallinity and a well-developed polycrystalline structure. In contrast, films deposited via SPVM exhibit lower peak intensities and a higher diffuse background. The dominance of selected diffraction peaks may indicate the presence of preferred orientation (texturing), rather than single-crystal growth. This behavior suggests a lower degree of structural ordering and/or the presence of fine-grained or partially disordered material [4]. These differences can be explained by the gas transport conditions in the two regimes. In SPVM, longer precursor residence time and limited gas flow create near-static conditions that may favor growth along preferred crystallographic directions. In DPVM, continuous gas flow and shorter residence time promote more homogeneous nucleation and more uniform polycrystalline structure with multiple crystallographic orientations [3,17]. As a result, DPVM leads to well-defined polycrystalline films, while SPVM tends to produce more disordered and/or preferentially oriented structures, regardless of the reactor volume. The AFM images with cross-sectional profiles with cross-sectional profiles presented in Figure 6 and Figure 7 further support the conclusions drawn from the XRD analysis. For the reactor with a volume of 470 cm^3^, both DPVM and SPVM films exhibit an RMS roughness of about 4 nm; however, the DPVM film shows a more homogeneous surface with finer and more uniformly distributed surface features, while the SPVM film contains a larger number of pronounced local surface protrusions. For the smaller reactor (47 cm^3^), the RMS roughness is approximately 4 nm for DPVM and about 3 nm for SPVM. Despite the slightly lower RMS value, the SPVM film exhibits a more diffuse and less well-defined surface texture, whereas the DPVM film maintains a more uniform and better-defined microstructure [20]. This indicates that RMS roughness alone is not sufficient to describe the surface quality and must be interpreted together with structural information from XRD and additional AFM-derived parameters (see Table 2). Importantly, reducing the reactor volume from 470 to 47 cm^3^ does not change the crystalline phase of ZnO and does not deteriorate the film quality. Instead, the positive effect of DPVM on crystallinity and microstructural uniformity is preserved in both reactor volumes.

**Figure 8:**
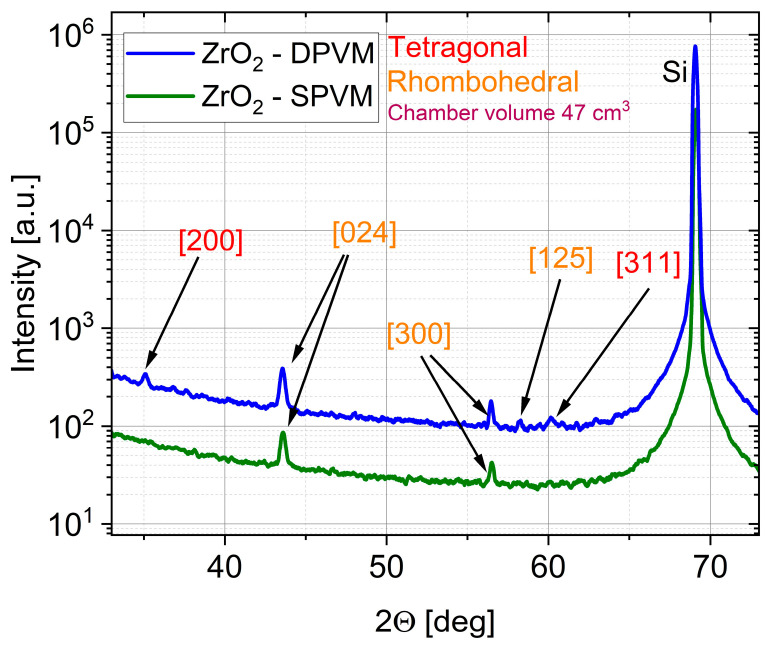
XRD patterns of ZrO_2_ thin films deposited at temperature lower than 100 °C by ALD in DPVM and SPVM in a reactor with a chamber volume of 47 cm^3^.

**Figure 9:**
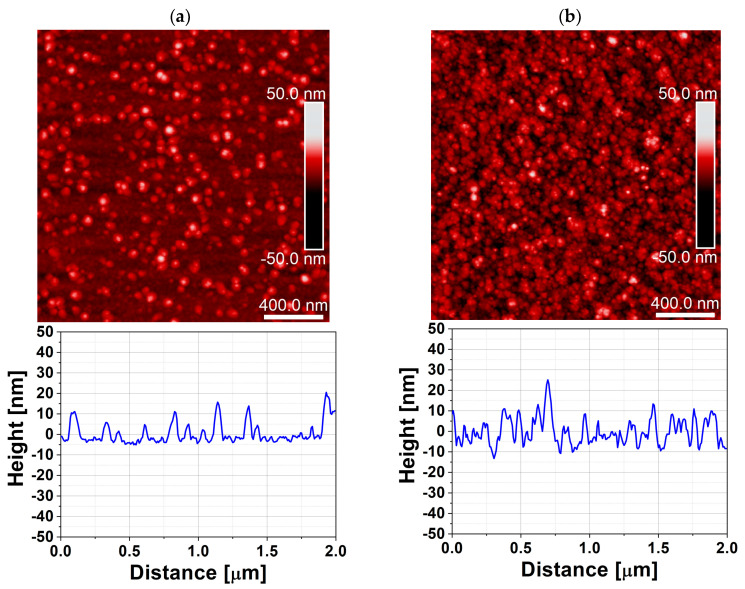
AFM surface topography with cross-sectional profile of ZrO_2_ thin films deposited at temperature lower than 100 °C by ALD in a reactor with a chamber volume of 47 cm^3^ using (**a**) dynamic purge vacuum mode (DPVM, RMS = 4 nm) and (**b**) static purge vacuum mode (SPVM, RMS = 6 nm). The scan area is 2 µm × 2 µm.

The effect of the purge mode is even more pronounced for ZrO_2_, which is known to be more sensitive to growth chemistry and process conditions than ZnO. For ZrO_2_ films deposited in the 47 cm^3^ chamber, the XRD patterns in Figure 8 reveal that the DPVM-deposited film exhibits distinctly higher diffraction intensities than the SPVM-deposited one. The observed reflections such as (200), (024), (300), (125), and (311) can be assigned to the tetragonal and rhombohedral phases of ZrO_2_, demonstrating that crystallization already occurs at this low temperature, particularly when DPVM is used. In contrast, the SPVM film shows much weaker diffraction peaks, indicating a lower degree of structural ordering. This is consistent with the AFM results with cross-sectional profiles in Figure 9, which show higher RMS roughness (6 nm) values for SPVM compared to DPVM (4 nm). The DPVM film exhibits a smoother and more homogeneous surface, which reflects a more uniform nucleation and growth process [20,21].

AFM parameters in Table 2 confirm that the AFM and XRD results are consistent and provide a coherent picture of the influence of the purge mode on film growth. For ZnO deposited in the 470 cm^3^ chamber, DPVM leads to a much higher particle density (0.17 µm^−2^) compared to SPVM (0.05 µm^−2^), while maintaining a similar RMS roughness of about 4 nm and a lower R_max_ (36 nm vs. 42 nm). This higher nucleation density correlates well with the XRD results, where the DPVM-deposited ZnO film exhibits significantly higher diffraction intensities. In the smaller 47 cm^3^ chamber, ZnO films deposited in DPVM also show a higher R_max_ (33 nm vs. 27 nm) and a lower particle density (0.10 µm^−2^ vs. 1.10 µm^−2^ in SPVM). Although the RMS roughness for the SPVM film (3 nm) is slightly lower than that obtained in DPVM (4 nm), the surface morphology appears more diffuse and less well-defined (Figure 7). The SPVM film exhibits less distinct grain features and a more irregular surface texture, indicating reduced microstructural uniformity [20]. In addition, the analysis of the threshold height and typical particle height/width parameters further supports the differences in the growth mechanism between DPVM and SPVM. For ZnO deposited in the 470 cm^3^ chamber, the typical particle dimensions remain similar (about 20 nm in height and 200 nm in width); however, the threshold height is lower for DPVM (25 nm) than for SPVM (32 nm). This indicates a narrower height distribution and a reduced contribution of tall, protruding surface features in the DPVM case. This observation is consistent with the lower R_max_ and higher particle density obtained for DPVM and explains the improved crystalline ordering observed in the XRD patterns. For ZrO_2_ deposited in the 47 cm^3^ chamber, the differences are even more pronounced: the RMS roughness increases from about 4 nm in DPVM to 6 nm in SPVM, and R_max_ increases from 35 nm to 54 nm, while the particle density rises from 15 to 24 µm^−2^, indicating a rougher and less homogeneous surface in SPVM. These morphological changes are directly reflected in the XRD patterns, where the DPVM film shows clearly higher diffraction intensities and better-defined peaks, evidencing improved crystallinity. Although the typical particle dimensions for ZrO_2_ (about 20–25 nm in height and 100 nm in width) and the threshold height (29 nm) are comparable, the SPVM film exhibits a much larger R_max_ (54 nm) compared to the DPVM film (35 nm). This indicates a larger contribution of high, non-uniform surface features and a less homogeneous growth mode in SPVM. Such morphological inhomogeneity directly correlates with the weaker and broader diffraction peaks observed in XRD, evidencing a lower degree of structural ordering.

To gain deeper mechanistic insight into the reaction kinetics, the relationship between reactor chamber volume and precursor dosing was analyzed. A significant finding of this study is the high kinetic efficiency observed during reactor scaling: while the effective chamber volume was increased tenfold (from 47 cm^3^ to 470 cm^3^), the optimum precursor pulse times required only a twofold increase to maintain full surface saturation. Crucially, all other growth parameters—including purging times after precursor pulses and deposition temperatures—were kept identical across both chamber reactor volumes and purge modes to ensure a rigorous comparison. The experimental data from both chamber volumes suggest that the structural integrity of the films is primarily governed by the gas transport regime. To support this interpretation, the characteristic gas residence time (τ) was considered using the standard relation τ = V/Q, where V is the reactor volume and Q is the volumetric gas flow rate [22]. According to this relation, a tenfold increase in chamber reactor volume should, in principle, lead to a proportional increase in residence time and thus require a correspondingly larger precursor pulse time to achieve saturation. However, the experimental results demonstrate that a mere twofold increase in precursor pulse time is sufficient. This indicates that surface saturation is not governed solely by the total chamber reactor volume or the bulk precursor concentration, but rather by the local availability of reactive species near the surface and the efficiency of mass transport [19]. The SPVM, applied consistently across both reactor sizes, introduces a 1 s ‘wait time’ with a closed stop valve. This interval results in a forced stagnation of the gas phase, extending the precursor residence time far beyond the limits of natural convective transport. This dwell time allows precursor molecules to linger and diffuse throughout the volume, which may promote undesirable gas-phase collisions or precursor re-adsorption. Such a mechanistic breakdown potentially explains the systematic differences observed in the XRD and AFM data. Specifically, the sharper diffraction peaks and more uniform surface textures in DPVM-grown films likely result from a continuous convective flow that effectively clears the precursor load. In contrast, the reduced crystallinity and local surface protrusions in SPVM-grown films—observed regardless of chamber size—might be a consequence of this forced stagnation, which could disrupt the ideal self-limiting ALD regime by creating conditions that may only resemble CVD-like processes [1,3].

Importantly, reducing the reactor chamber volume does not alter the phase composition of ZnO, but it preserves the positive effect of DPVM on structural quality. Altogether, the combined analysis of typical particle size, threshold height, RMS roughness, and R_max_ for ZnO and ZrO_2_ demonstrates that DPVM, even in the smaller reactor volume, promotes more uniform growth with a narrower height distribution and better-controlled microstructure, which is fully consistent with the improved crystallinity revealed by XRD. These results confirm that for both oxides, the purge mode is the dominant factor controlling the structural quality of the films. At the same time, the smaller reactor volume enables more efficient precursor utilization and enhanced control over gas-phase conditions without compromising film properties. Consequently, although SPVM yields a higher GPC than DPVM, this increase occurs at the expense of structural integrity and microstructural control. These observations confirm that DPVM facilitates a growth regime closer to ideal ALD through more effective purging and superior separation of the half-reactions. [3,17].

Crucially, the inferior uniformity and limited conformality inherent to the SPVM prevented the successful fabrication of functional optoelectronic devices, such as the phosphor and LED structures investigated here. We hypothesize that in SPVM-grown layers, a higher density of structural defects—including non-stoichiometric grain boundaries and localized pinholes—acts as potent non-radiative recombination centers and parasitic shunting paths [23]. These defects lead to excessive leakage currents and thermal instability, effectively rendering SPVM films unsuitable for integration into active optoelectronic architectures [24]. In contrast, the microstructural control afforded by DPVM suppresses these loss mechanisms, providing the film quality necessary for functional device integration.

Consequently, the following sections focus on demonstrating the feasibility of integrating these optimized DPVM-grown layers into phosphor and LED structures, as this regime ensures the structural integrity required for functional optoelectronic performance.

Although low-temperature ALD, especially in DPVM, already provides good uniformity and high conformality, higher growth temperatures remain preferable for device-quality layers [4,17]. At elevated temperatures, atoms on the growing surface can more easily rearrange into energetically favorable lattice positions, which promotes grain growth, reduces defects, and improves crystallinity and microstructural stability [4]. These factors are critical for electronic and optoelectronic devices, where structural defects act as charge traps and scattering centers, degrading carrier transport, increasing leakage currents, and reducing optical efficiency. Moreover, films grown at higher temperature typically exhibit better thermal and electrical stability, which is essential for reliable long-term operation of devices such as n-i-p heterostructure LEDs and phosphor-based structures. Therefore, while low-temperature processes are indispensable for integration with temperature-sensitive substrates and for achieving highly conformal coatings, higher growth temperatures are still preferred whenever the highest structural quality and device performance are required [4,17].

**Figure 10:**
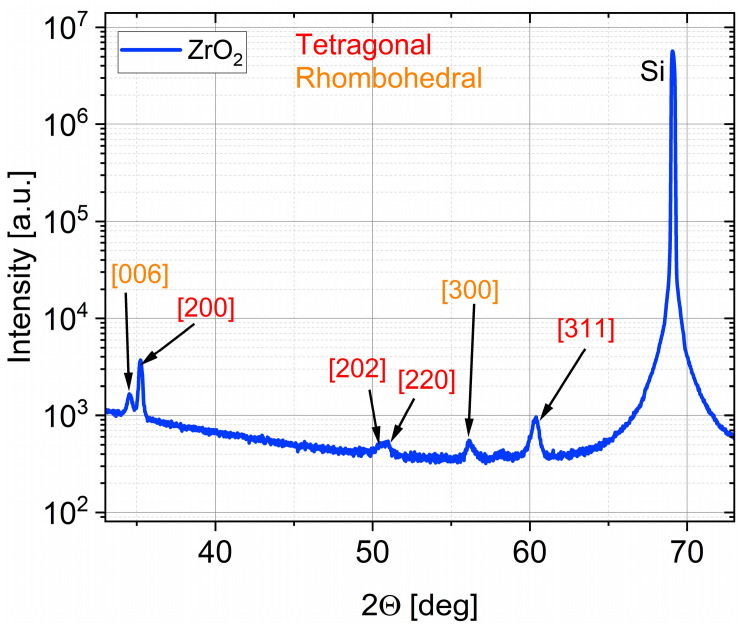
XRD pattern of a ZrO_2_ film deposited at temperature of 240 °C (DPVM, 47 cm^3^ reactor), showing tetragonal and rhombohedral phases.

**Figure 11:**
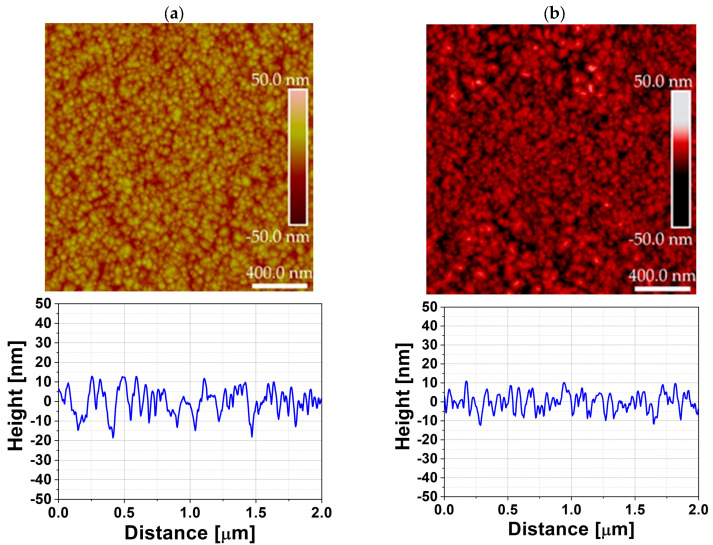
AFM surface topography with cross-sectional profile of ZrO_2_: RE thin films deposited at temperature of 240 °C by ALD (DPVM, 47 cm^3^ reactor): (**a**) as-grown films (RMS = 7 nm) and (**b**) films after annealing at 800 °C (RMS = 5 nm). The scan area is 2 µm × 2 µm.

The XRD pattern in Figure 10 of the ZrO_2_ film deposited at 240 °C in DPVM in the 47 cm^3^ chamber confirms the presence of tetragonal and rhombohedral phases, indicating a high degree of crystallization already during growth. Well-defined reflections such as (006), (200), (202), (220), (300), and (311) with relatively high intensities demonstrate good structural quality and a low contribution of any amorphous phase. This indicates that the elevated growth temperature effectively promotes structural ordering of the ZrO_2_ lattice. The obtained well-defined and crystalline ZrO_2_ structure provides a suitable host matrix for the incorporation of rare-earth (RE) ions such as europium (Eu), terbium (Tb), and Cerium (Ce). The preserved phase purity and absence of secondary phases suggest that such a structurally ordered lattice is highly promising for rare-earth doping without significantly disturbing the crystal framework, as reported in previous studies [25]. AFM analysis presented in Figure 11 and Table 3 shows that the as-grown ZrO_2_:RE film exhibits an RMS roughness of about 7 nm, which decreases to approximately 5 nm after annealing at 800 °C. At the same time, the typical particle height and width remain around 20 nm and 100 nm, respectively. This is accompanied by a slight reduction in R_max_ (from 50 nm to 49 nm) and a decrease in threshold height (from 22 nm to 19 nm), pointing to a more uniform height distribution of surface features. The particle density remains nearly unchanged (from 99 to 91 µm^−2^), suggesting that thermal treatment mainly promotes grain growth and surface smoothing rather than renucleation. Altogether, these results demonstrate that high-temperature growth and post-deposition annealing improve the structural and morphological quality of the ZrO_2_ host and provide more favorable conditions for the activation of RE-related luminescent centers, which is crucial for phosphor and optoelectronic applications [20,25].

**Figure 12:**
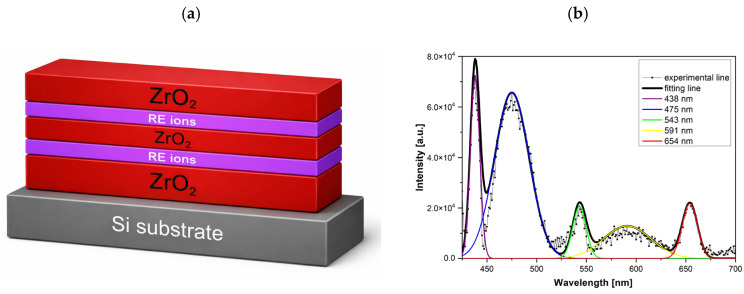
(**a**) Schematic diagram of the multilayer ZrO_2_:RE phosphor structure deposited at temperature 240 °C on a silicon substrate. (**b**) Photoluminescence (PL) spectrum of the ZrO_2_:RE thin films after annealing at 800 °C together with multi-peak Gaussian fitting, showing emission bands centered at approximately 438, 475, 543, 591, and 654 nm attributed to different defect-related radiative recombination channels.

As mentioned in our previous work [12], the schematic of the multilayer ZrO_2_:RE structure shown in Figure 12a illustrates a phosphor architecture consisting of alternating ZrO_2_ layers and RE-doped active layers deposited on a silicon substrate, where the rare-earth ions are Eu, Tb, and Ce. Such multilayer systems require both precise thickness control and high structural quality, since the optical activity of RE ions is strongly influenced by the crystallinity of the host matrix and the local structural environment. The photoluminescence spectrum in Figure 12b of the ZrO_2_:RE films after annealing at 800 °C, decomposed using multi-peak Gaussian fitting, reveals emission bands centered at approximately 438, 475, 543, 591, and 654 nm. These emission bands are commonly associated with defect-related states in the ZrO_2_ host as well as with optical centers related to rare-earth ions [25,26,27]. Given the complexity of the defect structure of ZrO_2_ and the possible overlap of emission bands, a unique assignment of each band to a specific RE ion (Eu, Tb, or Ce) is not straightforward. Nevertheless, the presence of RE dopants clearly modifies the emission spectrum and confirms the optical activity of the ZrO_2_:RE system after high-temperature annealing.

**Figure 13:**
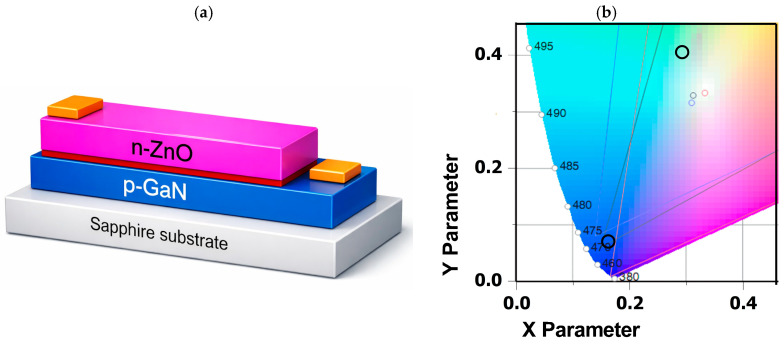
(**a**) Schematic cross-section of the n-ZnO/i-layer/p-GaN heterostructure diode deposited on a sapphire substrate, featuring ohmic metallic contacts. The intrinsic layer (i-layer) and the ohmic contacts are highlighted in red and orange, respectively. (**b**) CIE chromaticity diagram for the n-ZnO/Al_2_O_3_/HfO_2_/p-GaN heterostructure light-emitting diodes. The black circles represent the diode emission coordinates, confirming that our n-i-p structures enable efficient color tuning from blue emission toward visible white light. The n-ZnO deposited at temperature 300 °C by ALD (DPVM, 47 cm^3^ reactor) on p-GaN.

**Figure 14:**
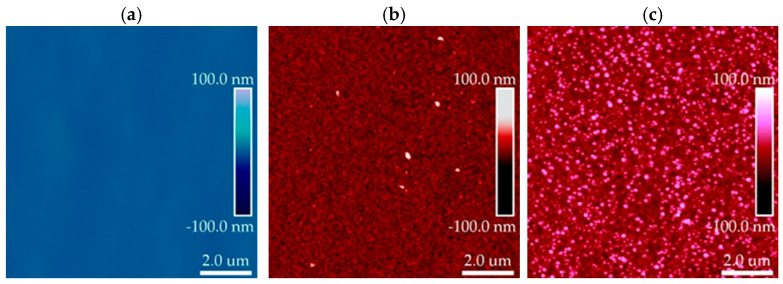
AFM surface topography of (**a**) p-GaN substrate (RMS = 1 nm), (**b**) p-GaN/i/n-ZnO structure (RMS = 7 nm), and (**c**) p-GaN/n-ZnO structure (RMS = 16 nm). The scan area is 10 µm × 10 µm. The insertion of the intrinsic interlayer reduces surface roughness and leads to a more homogeneous surface morphology compared to direct n-ZnO deposition at temperature 300 °C by ALD (DPVM, 47 cm^3^ reactor) on p-GaN.

As mentioned in our previous work [13], Figure 13 illustrates the device concept and its optical response. The schematic cross-section shows the n-ZnO/i-layer/p-GaN heterostructure diode deposited on a sapphire substrate, where both the ZnO layer and the intrinsic interlayer were grown by ALD in DPVM in a reactor with an effective chamber volume of 47 cm^3^. As demonstrated in the previous sections, the DPVM process provides a growth regime closer to ideal, self-limited ALD, with improved microstructural control compared to SPVM, as evidenced by the combined AFM and XRD analyses. This is particularly important for device fabrication, where smooth, uniform, and highly conformal layers are required. The corresponding CIE chromaticity diagram for the n-ZnO/Al_2_O_3_/HfO_2_/p-GaN heterostructure diodes confirms that our n-i-p structures enable efficient color tuning from blue emission toward visible white light. In agreement with the earlier structural analysis, ZnO layers grown at the higher temperature of 300 °C exhibit improved crystallinity and a well-defined wurtzite structure, which directly translates into more stable and reproducible optical emission [6,23]. For these films, the emission coordinates fall within the cold-white region of the CIE diagram, demonstrating the potential of this approach for white-light optoelectronic applications. AFM images in Figure 14 further support the conclusions drawn from the earlier morphological analysis. The insertion of the intrinsic interlayer reduces the surface roughness from 16 nm for the p-GaN/n-ZnO structure to 7 nm for the p-GaN/i/n-ZnO structure, while the bare p-GaN substrate shows an RMS value of about 1 nm. This behavior is fully consistent with the previously discussed role of growth mode and process control: smoother and more homogeneous interfaces are obtained when growth proceeds in a well-controlled ALD regime, which is essential for minimizing interface-related defects in device structures. Finally, ZnO films grown at the relatively higher temperature of 300 °C form hexagonal columnar structures and exhibit the wurtzite phase, as evidenced by the XRD reflections (10.0), (00.2), (10.1), and (11.0), in agreement with our previous reports [7].

This observation directly links the earlier structural discussion with device performance: higher growth temperature improves crystallinity and microstructural ordering, while DPVM ensures high conformality and uniformity. Together, these factors explain why the optimized ALD process conditions lead not only to improved film quality but also to superior and tunable electrical and optical responses of ZnO- or ZrO_2_-based device structures.

## 4. Conclusions

This work establishes the ALD purge mode as a critical factor governing the growth behavior of ZnO and ZrO_2_ thin films. The results indicate that purge-mode engineering provides a powerful, cost-effective route to control film microstructure and crystallinity without altering the precursor chemistry or growth temperature. Although the static purge vacuum mode provides a higher growth per cycle, it results in less controlled growth due to extended precursor residence times which increase the probability of re-adsorption and may promote secondary surface and gas-phase interactions.

In contrast, the dynamic purge vacuum mode ensures a growth regime closer to ideal ALD conditions, leading to smoother surfaces, lower R_max_ and threshold height values, and improved crystallinity, as confirmed by AFM and XRD analyses. No significant differences in GPC were observed between reactors with different chamber volumes, which justifies the use of the smaller chamber to enable more efficient and uniform deposition with reduced precursor consumption. For ZrO_2_, DPVM enables the formation of crystalline tetragonal and rhombohedral phases already at low temperatures (below 100 °C), while higher growth temperatures (above 200 °C) and post-deposition annealing further enhance crystallinity and microstructural stability. For ZnO, DPVM improves microstructural control, and growth at elevated temperature (300 °C) results in a well-defined wurtzite structure with a hexagonal columnar morphology. Overall, the combination of DPVM and optimized growth temperature provides a robust route for fabricating good quality, highly conformal ZnO and ZrO_2_ thin films suitable for advanced oxide-based structures, such as phosphors and n-i-p light-emitting diodes.

## Figures and Tables

**Table 1:** Growth per cycle for ZnO and ZrO_2_ thin films deposited by ALD at temperature lower than 100 °C with 47 and 470 cm^3^ effective chamber volumes using dynamic and static purge vacuum modes.

Material	Chamber Volume [cm^3^]	Growth Per Cycle for DPVM [Å]	Growth Per Cycle for SPVM [Å]
ZnO	470	1.0	1.3
ZnO	47	1.0	1.3
ZrO_2_	47	1.4	1.6

**Table 2:** Summary of surface morphology parameters obtained from AFM analysis for ZnO and ZrO_2_ thin films deposited by ALD using dynamic purge vacuum mode (DPVM) and static purge vacuum mode (SPVM) in reactors with different effective chamber volumes. The table lists RMS roughness, typical particle height/width, maximum height difference (R_max_), threshold height, and particle density.

Material	Chamber Volume [cm^3^]	DPVM					SPVM				
		RMS [nm]	Typical Particle Height/ Width [nm]	R_max_ [nm]	Threshold Height [nm]	Density [Particle Number/µm^2^]	RMS [nm]	Typical Particle Height/ Width [nm]	R_max_ [nm]	Threshold Height [nm]	Density [Particle Number/µm^2^]
ZnO	470	4	~20/200	36	25	0.17	4	~20/200	42	32	0.05
ZnO	47	4	~15/200	33	24	0.1	3	~10/200	27	14	1.1
ZrO_2_	47	4	~20/100	35	29	15	6	~25/100	54	29	24

**Table 3:** Summary of AFM-derived surface morphology parameters of ZrO_2_ thin films deposited by ALD in dynamic purge vacuum mode (DPVM) in a reactor with an effective chamber volume of 47 cm^3^ at a growth temperature of 240 °C, for the as-grown film and after annealing at 800 °C. The table lists RMS roughness, typical particle height and width, maximum height difference (R_max_), threshold height, and particle density.

Growth Temperature [°C]	Annealing Temperature [°C]	RMS [nm]	Typical Particle Height on the Surface [nm]	Typical Particle Width on the Surface [nm]	R_max_ [nm]	Threshold Height [nm]	Density [Particle Number/µm^2^]
240	as grown	7	~20	~100	50	22	99
	800	5	~20		49	19	91

## Data Availability

The original contributions presented in this study are included in the article. Further inquiries can be directed to the corresponding author.
